# Evidence-based assessment of antiosteoporotic activity of petroleum-ether extract of *Cissus quadrangularis* Linn. on ovariectomy-induced osteoporosis

**DOI:** 10.1080/03009730902891784

**Published:** 2009-09-07

**Authors:** Bhagath K Potu, Muddanna S Rao, Gopalan K Nampurath, Mallikarjuna R Chamallamudi, Keerthana Prasad, Soubhagya R Nayak, Praveen K Dharmavarapu, Vivekananda Kedage, Kumar MR Bhat

**Affiliations:** ^1^Department of Anatomy, Kasturba Medical College, Manipal UniversityManipal, Karnataka, 576104India; ^2^Department of Pharmacology, Manipal College of Pharmaceutical SciencesManipal, Karnataka, 576104India; ^3^Manipal Centre of Information Sciences, Manipal UniversityManipal, Karnataka, 576104India; ^4^Department of Anatomy, Centre for Basic Sciences, Kasturba Medical CollegeMangalore, Karnataka, 575004India; ^5^Department of Biochemistry, Kasturba Medical College, Manipal UniversityManipal, Karnataka, 576104India; ^6^Department of Anatomy, Kasturba Medical College, Manipal UniversityManipal, Karnataka, 576104India

**Keywords:** Antiosteoporotic agents, ovariectomy, phytogenic steroid, postmenopausal osteoporosis

## Abstract

The increasing incidence of postmenopausal osteoporosis and its related fractures have become global health issues in the recent days. Postmenopausal osteoporosis is the most frequent metabolic bone disease; it is characterized by a rapid loss of mineralized bone tissue. Hormone replacement therapy has proven efficacious in preventing bone loss but not desirable to many women due to its side-effects. Therefore we are in need to search the natural compounds for a treatment of postmenopausal symptoms in women with no toxic effects. In the present study, we have evaluated the effect of petroleum-ether extract of *Cissus quadrangularis* Linn. (CQ), a plant used in folk medicine, on an osteoporotic rat model developed by ovariectomy. In this experiment, healthy female Wistar rats were divided into four groups of six animals each. Group 1 was sham operated. All the remaining groups were ovariectomized. Group 2 was fed with an equivolume of saline and served as ovariectomized control (OVX). Groups 3 and 4 were orally treated with raloxifene (5.4 mg/kg) and petroleum-ether extract of CQ (500 mg/kg), respectively, for 3 months. The findings were assessed on the basis of animal weight, morphology of femur, and histochemical localization of alkaline phosphatase (ALP) (an osteoblastic marker) and tartrate-resistant acid phosphatase (TRAP) (an osteoclastic marker) in upper end of femur. The study revealed for the first time that the petroleum-ether extract of CQ reduced bone loss, as evidenced by the weight gain in femur, and also reduced the osteoclastic activity there by facilitating bone formation when compared to the OVX group. The osteoclastic activity was confirmed by TRAP staining, and the bone formation was assessed by ALP staining in the femur sections. The color intensity of TRAP and ALP enzymes from the images were evaluated by image analysis software developed locally. The effect of CQ was found to be effective on both enzymes, and it might be a potential candidate for prevention and treatment of postmenopausal osteoporosis. The biological activity of CQ on bone may be attributed to the phytogenic steroids present in it.

## Introduction

A substantial body of evidence indicates that osteoporosis is a global health problem characterized by the microarchitectural deterioration of bone mass and strength that leads to fragility fractures, and it is occurring in every geographic area, affecting 150 million people worldwide. The most effective treatment for the prevention of osteoporosis is hormone replacement therapy. However, because of an increased risk of breast cancer and an unacceptable rate of undesirable outcomes, a large-scale clinical intervention study was terminated early on the advice of the Data and Safety Monitoring Board in the United States (1). For quite a long time, estrogen, bisphosphonates, calcitonin, calcium products, ipriflavone (2), and anabolic steroids have been used clinically as effective medications. However, each one of them is associated with numerous side-effects (3,4). So active attempts still are being vigorously pursued to identify new inhibitors of bone resorption that minimize the necessity for drug therapy (4). Traditional medicine has been widely used for thousands of years to treat bone disorders. Although these herbal medicines are seen as cost-effective alternatives by their traditional users, their international acceptance as a major regimen for prevention and treatment of osteoporosis would require extensive research using modern science. As a part of our continuing screening of biologically active antiosteoporotic agents, we made an attempt to evaluate the effect of petroleum-ether extract of *Cissus quadrangularis* Linn. (CQ) on osteoporosis. CQ (Veldt Grape or Winged Treebine), a climber in the family Vitaceae, is one of the most frequently used medicinal plants in India and can be found throughout the country (5). The fresh stem and leaves of CQ are used for the treatment of various ailments (6–22). Pharmacological studies have revealed the bone fracture healing property (7,8) and antiosteoporotic effect of this plant (13). Shirwaikar et al. found that 750 mg/kg of body-weight of ethanolic extract given to rats was effective in ovariectomy-induced osteoporosis (13). Previous studies from our laboratory reported the effects of ethanolic and petroleum-ether extract of CQ on the ossification of fetal bone (23–25).

In our quest to identify and isolate the active principles of the CQ extract, we have more effectively extracted specific chemical compounds from the plant using different organic solvents and have tested these extracts for their osteostimulant role *in vitro*. We have observed that CQ petroleum-ether extract has a pronounced effect on osteoblasts (unpublished data). It was therefore pertinent to study the antiosteoporotic activity of petroleum-ether extract of CQ, which has not been previously reported.

## Materials and methods

### Plant material and extraction

The stem of *Cissus quadrangularis* plant was collected in the Nalgonda District of Andhra Pradesh, India, identified and authenticated by a botanist, and a voucher specimen was deposited in the pharmacology department of the Manipal University, Manipal. The fleshy stems (2.5 kg) were washed, cut into small pieces, air-dried, and crushed into powder. Stem powder was exhaustively extracted with 95% ethanol using a Soxhlet apparatus. A yield of 225 g was obtained. The total ethanol extract was concentrated in vacuum, the extract was dissolved in water, and the solution was partitioned with petroleum-ether to obtain petroleum-ether extract in a yield of 18.2 g.

### Animals

A total of 114 (90 of both sexes, and 24 female) 3-month-old Wistar rats weighing approximately 225 g were housed in the central animal research facility of Manipal University. The rats were housed in sanitized polypropylene cages containing sterile paddy husk as bedding. The animals were maintained under controlled conditions of temperature (23±2°C), humidity (50%±5%), and a 12-h light–dark cycle. All animals were allowed free access to distilled water and fed on a commercial diet. All the studies conducted were approved by the Institutional Animal Ethical Committee (No. IAEC/KMC/06/2006–2007), Kasturba Medical College, Manipal, according to prescribed guide-lines of the Committee for the Purpose of Control and Supervision of Experiments on Animals (CPCSEA), Government of India.

### Acute toxicity studies

Acute toxicity was determined in fasting rats. Animals were divided into groups of 10 each and given orally 500, 1000, 1500, 2500, 3000, 3500, 4000, 4500, and 5000 mg/kg body-weight (bw) of CQ. The rats were observed continuously for 2 h, then, frequently up to 6 hours, and daily thereafter for 30 days, and mortality if any was recorded (26).

### Experimental protocol

After 7 days of acclimatization, the female Wistar rats (24 in number) were ovariectomized or sham operated. The rats were anesthetized with pentobarbital sodium (40 mg/kg bw, intra peritoneally(i.p.)), and the ovaries were removed bilaterally. Sham operation was performed in the same manner but only exposing the ovaries (sham-operated (SHAM) group). A week later, the ovariectomized rats were randomly divided into 3 groups of six animals each. The groups are: 1) sham operated (SHAM), 2) ovariectomized (OVX), 3) ovariectomized and treated with 5.4 mg/kg bw of raloxifene (OVX + RAL), and 4) ovariectomized and treated with 500 mg/kg bw of petroleum-ether extract of CQ (OVX + CQ). The treatment continued for 3 months. At the end of the 3 months of treatment, rats in all groups were weighed and sacrificed by cervical dislocation and femur bones were dissected.

### Measurement of femur length and weight

The femur length was measured as the distance between the greater trochanter and the medial condyle (27) in right femurs using vernier calipers. The same femurs were then dried in an oven at 110°C for 48 h and weighed using a digital weighing device.

### Tissue processing for alkaline phosphatase (ALP), and tartrate-resistant acid phosphatase (TRAP) staining

The left femurs were removed, dissected free of soft tissue, and fixed with periodate-lysine-paraformaldehyde (PLP) fixative containing 2% paraformaldehyde, 0.075 M lysine, and 0.01 M sodium periodate solution (pH 7.4, stored at 4°C) for 24 h at 4°C as described previously (28). The bones were decalcified by the protocol described previously (29,30). Thereafter tissues were dehydrated in a graded series of alcohols and embedded in paraffin wax. Care was taken to ensure that all bones were oriented in the same direction during embedding to minimize differences in the angles at which the bones were sectioned, and a single investigator processed all bones to maintain consistency. The upper ends of femurs were cut longitudinally at 5 µm thickness in a rotary microtome and processed for alkaline phosphatase (ALP) and tartrate-resistant acid phosphatase (TRAP) staining.

### Histochemical localization of alkaline phosphatase (ALP)

Bone sections were deparaffinized with xylene and hydrated through graded alcohol series. The sections were preincubated overnight in 1% magnesium chloride in 100 mL Tris-maleate buffer (pH 9.2) and then incubated for 2 h at room temperature in ALP substrate solution (freshly prepared 100 mM Tris-maleate buffer, pH 9.2, containing 0.2 mg/mL naphthol AS-MX phosphate (Sigma, USA) and 0.4 mg/mL Fast Red TR (Sigma)). After washing with distilled water, the sections were counterstained with Vector methyl green nuclear counterstain (Vector Laboratories, Peterborough, UK) and mounted with Kaiser's glycerol jelly (31). Stained sections were photographed with a digital camera attached to a binocular microscope. These digital images were analyzed for ALP color intensity using image analysis software developed locally.

### Histochemical localization of tartrate-resistant acid phosphatase (TRAP)

The sections were deparaffinized with xylene and preincubated for 20 minutes in sodium acetate (50 mM) and potassium sodium tartrate (40 mM) buffer (pH 5.0) and then incubated for a further 15 min at room temperature in the same buffer containing naphthol AS-MX phosphate (2.5 mg/mL) (Sigma) and fast garnet GBC (0.5 mg/mL) (Sigma). After washing with distilled water, the sections were counterstained with Vector methyl green nuclear counterstain (Vector laboratories, Peterborough, UK) and mounted in Kaiser's glycerol jelly (32). Stained sections were photographed with a digital camera attached to a binocular microscope. These digital images were analyzed for TRAP color intensity using image analysis software developed locally.

### Image analysis

The images were analyzed using the in-house-developed software named Tissue Quant 1.0. The facility for choosing the particular color representing the maximum density of tissue as a reference color is provided in the software. Using this facility, the color setting was done for the analysis purpose. The same setting was used for the analysis of all the images.

The software assigns scores to the various shades of the color represented by each pixel of the image, based on how close the shade is to the reference color. Using these values the total tissue present in the image is quantified (33).

### Statistical analysis

The results were expressed as the mean with the standard error of mean. All data were analyzed using Graphpad Prism software (Microsoft, San Diego, CA, USA). One-way ANOVA was first performed to test for any significant difference among the groups. When significant, a post-test, Bonferroni's multiple comparison test was applied to determine the specific difference between the groups. The level of significance was *P*<0.05 for all tests.

## Results

Administration of the plant extract did not present any adverse effect on the treated animals. All animals were healthy throughout the experimental period.

### Effect of CQ on body-weight

**Figure 1.**
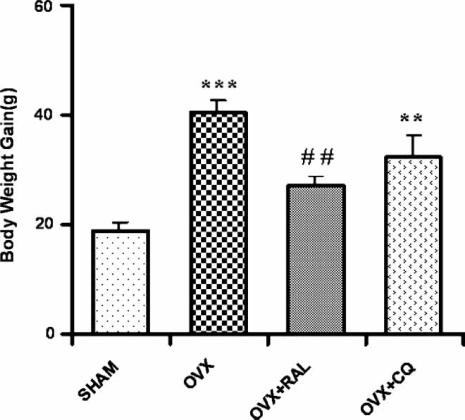
Body-weight gain in sham control (SHAM), ovariectomized (OVX), ovariectomized and raloxifene treated (OVX + RAL), and ovariectomized and *Cissus quadrangularis* extract treated (OVX + CQ) groups during 3 months treatment period. Note the significant increase in the body-weight gain in OVX when compared to SHAM group (***P<0.001). The body-weight is significantly decreased when the OVX animals were treated with CQ (**P<0.01) and raloxifene (##*P*<0.01), respectively.

The effect of CQ on the body-weights of all the groups is presented in Figure 1. There was a significant difference between the SHAM, OVX, OVX + RAL, and the OVX + CQ-treated groups at the end of the experimental period. The OVX rats had a significant weight increase compared with SHAM animals (*P*<0.001). The weight decreased significantly compared with the OVX group after treatment with raloxifene and CQ (*P*<0.01). With respect to food consumption the animals in all the groups had similar food.

### Effect of CQ on femur weight and length

Ovariectomy resulted in a significant reduction in femoral weight when compared with SHAM group (*P*<0.001). Treatment with raloxifene and CQ significantly increased the femoral weight and brought it closer to the SHAM group (*P*<0.001, Table I). There was a slight change in the femoral length among the groups Table I

**Table I.** Femur weight and length. Femur weight and length in sham control (SHAM), ovariectomized (OVX), ovariectomized and raloxifene-treated (OVX + RAL), and ovariectomized and *Cissus quadrangularis* extract-treated (OVX + CQ) groups. Note there is a significant decrease in the femur weight in the OVX group compared to the SHAM group and a significant increase in the femur weight in the OVX + RAL and OVX + CQ groups compared to the OVX Group. Also note that there is a slight increase in the femur length in the OVX compared to the SHAM group and a significant decrease in the femur length in the OVX + RAL and OVX + CQ groups compared to the OVX Group.

Group	Femur weight (g)	Femur length (mm)
SHAM	0.49±0.008	31.6±0.170
OVX	0.37±0.008^a^	32.7±0.307^c^
OVX + RAL	0.44±0.003^b^	31.8±0.147^c^
OVX + CQ	0.44±0.003^b^	31.8±0.147^c^

^a^*P*<0.001. ^b^*P*<0.001, one-way ANOVA, Bonferroni's test. ^c^*P <*0.01.

### Effect of CQ on alkaline phosphatase activity in the bone sections

**Figure 2.**
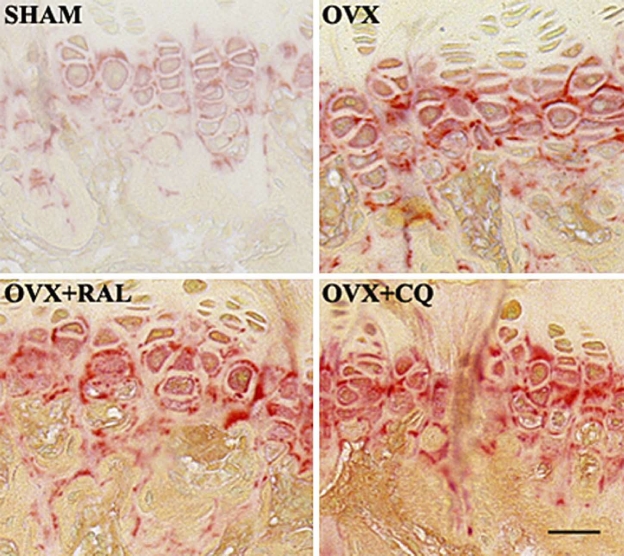
Histochemical localization of alkaline phosphatase in bone sections of sham control (SHAM), ovariectomized (OVX), ovariectomized and raloxifene-treated (OVX + RAL) and ovariectomized and treated with *Cissus quadrangularis* extract (OVX + CQ) groups. Note the significant increase in the staining intensity in OVX, OVX + RAL, OVX + CQ groups compared to SHAM group.

**Figure 3.**
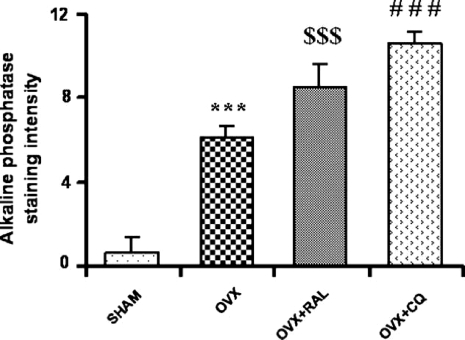
Alkaline phosphatase staining intensity score in bone sections, measured using the software in sham control (SHAM), ovariectomized (OVX), ovariectomized and raloxifene-treated (OVX + RAL), and ovariectomized and treated with *Cissus quadrangularis* plant extract (OVX + CQ) groups. SHAM versus OVX: ***P<0.001; SHAM versus OVX + RAL: $$$*P*<0.001; SHAM versus OVX + CQ: ###*P*<0.001.

The results of histochemical localization of ALP of different groups studied are shown in Figures 2 and 3. As compared to group 1, group 2 animals showed a significant rise in ALP levels (*P*<0.001) at the end of 3 months following ovariectomy. Group 3 and 4 showed a further increase in ALP levels following administration of raloxifene and CQ (*P*<0.001).

### Effect of CQ on tartrate-resistant acid phosphatase activity in the bone sections

**Figure 4.**
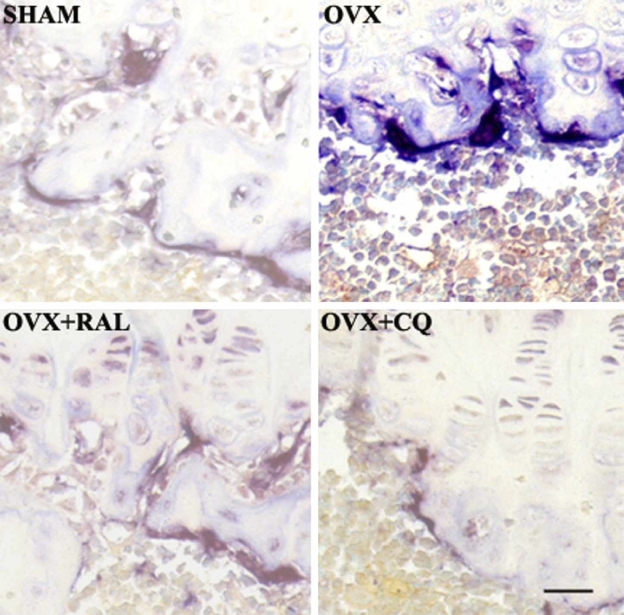
Histochemical localization of tartrate-resistant acid phosphatase (TRAP) in bone sections of sham control (SHAM), ovariectomized (OVX), ovariectomized and raloxifene-treated (OVX + RAL) and ovariectomized and *Cissus quadrangularis* extract-treated (OVX + CQ) groups. Note the increased activity of TRAP in the OVX Group.

**Figure 5.**
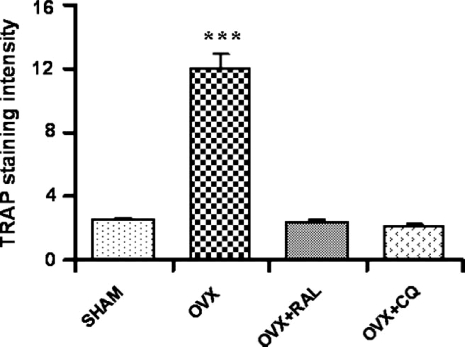
Tartrate-resistant acid phosphatase (TRAP) staining intensity score in bone sections measured using the software in Sham control (SHAM), ovariectomized (OVX), ovariectomized and raloxifene-treated (OVX + RAL), and ovariectomized and *Cissus quadrangularis* extract-treated (OVX + CQ) groups. SHAM versus OVX: ***P<0.001 (one-way ANOVA, Bonferroni's test).

The results of histochemical localization of TRAP of different groups studied are shown in Figures 4 and 5. As compared to group 1, group 2 animals showed a significant rise in TRAP levels (*P*<0.001) at the end of 3 months following ovariectomy. Group 3 and 4 showed a significant decrease in TRAP levels followed by administration of raloxifene and CQ.

## Discussion

The current approach to the design of antiosteoporotic drugs is directed along the two basic processes of bone remodeling. These are agents aimed at preventing bone resorption (estrogen, calcitonin, bisphosphonates, calcium, vitamin D, raloxifene) and agents that stimulate bone formation (fluoride, anabolic steroids) (34). Among these, estrogen replacement theraphy (ERT) used to be a popular regime for prevention and treatment of postmenopausal osteoporosis. However, recent evidence suggests that ERT is associated with increased risk of breast, ovarian, and endometrial cancer (35,36). In addition, the most frequently used antiosteoporotic drugs are developed in affluent countries, and the costs are too high to benefit the ordinary people in developing or even developed countries. Thus, alternative approaches for managing osteoporosis are needed.

The present study is the first to evaluate the effect of petroleum-ether extract of CQ on osteoporosis induced by ovariectomy. It is well known that estrogen deficiency is an important risk factor in the pathogenesis of osteoporosis. Our present study clearly demonstrated the usefulness and beneficial effects of CQ in the treatment of osteoporosis induced by ovariectomy. Estrogen influences bone loss either directly by binding to the receptor on the bone or indirectly by influencing calcium regulatory hormones (parathyroid hormone (PTH) and vitamin D) and cytokines interleukin (IL)-1 and IL-6. Ovariectomy results in a body-weight gain and a drastic decrease in bone mineral density. These changes are mostly due to estrogen deficiency.

The animals in the OVX group had a greater body-weight gain compared with the other groups. This weight gain due to ovariectomy despite similar food consumption has been well documented (37). Treatment with CQ and raloxifene reduced the body-weight and increased the bone mass when compared with the SHAM and the OVX animals; this finding is of particular significance because it may be due to the stimulatory effect of CQ on the synthesis of growth hormone (GH), which is known to decrease the adipose tissue mass (38) and to increase the bone mass (39). A slight increase in femoral length could be observed in the OVX group compared with the other groups, indicating the effect of estrogen deficiency on the longitudinal growth (40).

Our results clearly indicated that CQ extract could suppress bone resorption induced by estrogen deficiency. Tartrate-resistant acid phosphatase (TRAP) is the most widely recognized marker for bone resorption. Our data on histochemical localization of TRAP supported that CQ and raloxifene administration prevents elevation of TRAP occurring due to ovarian hormone deficiency.

Alkaline phosphatase is an enzyme present in many tissue cells like liver, kidney, intestine, placenta, and germ cells and in osteoblasts. Bone ALP has become the clinically most relevant enzyme in the diagnosis of bone diseases. The ALP activity on sections was seen more in the OVX group, due to lack of inhibiting activity of estrogen on osteoclasts causing the increase in bone resorption. Simultaneously with intensification of resorption process, the bone formation process was also increased by enhancement of osteoblast activity. It led to growth of ALP activity in the OVX group (41), whereas in CQ- and raloxifene-treated groups the ALP activity was seen more than OVX. Since petroleum-ether extract of CQ improved the activity of alkaline phosphatase, the messenger initiating calcification, it is possible that the herb accelerated mineralization of the organic matrix, thus speeding up the bone formation.

To our knowledge, the biological activity of CQ on bone may be due to one or more of the phytogenic steroids isolated, which are believed to be the main constituent of CQ. Studies on fracture healing suggested that the unidentified anabolic steroid isolated by Sen (42) acts on estrogen receptors of the bone cells. It also contains flavonoids, triterpenoids (43–45) stilbene derivatives, and many others, e.g. resveratrol, piceatannol, pallidol, parthenocissine (46–48), and phytosterols (49). However, it remains unknown which particular ingredient has effect on the above variables studied. With all the above facts, the antiosteoporotic activity of petroleum-ether extract of CQ may justifiably be attributed to the steroids present in it, which probably act as phytoestrogens to prevent the bone loss (50).

## Conclusion

The results obtained in the present study provide evidence that CQ contributes importantly to the prevention of bone loss induced by ovariectomy in rats. However, in order to develop CQ extract in the international scientific community as an alternative regime for the treatment of bone diseases, more research will be needed to identify the active ingredients in CQ extract as well as the mechanism that mediates the action of CQ extract *in vivo*.
